# Development of New Drugs for an Old Target — The Penicillin Binding Proteins

**DOI:** 10.3390/molecules171112478

**Published:** 2012-10-24

**Authors:** Astrid Zervosen, Eric Sauvage, Jean-Marie Frère, Paulette Charlier, André Luxen

**Affiliations:** 1 Centre de Recherches du Cyclotron, B30, Université de Liège, Sart-Tilman, B-4000 Liège, Belgium; Email: aluxen@ulg.ac.be; 2 Centre d’Ingénerie des Proteines, Institut de Chimie, B6a, Université de Liège, Sart-Tilman, B-4000 Liège, Belgium; Email: eric.sauvage@ulg.ac.be (E.S.); JMFrere@ulg.ac.be (J.-M.F.); Paulette.Charlier@ulg.ac.be (P.C.)

**Keywords:** non-β-lactam, penicillin binding protein, β-lactam resistance, transition state analogs, substrate analogs, inhibitors

## Abstract

The widespread use of β-lactam antibiotics has led to the worldwide appearance of drug-resistant strains. Bacteria have developed resistance to β-lactams by two main mechanisms: the production of β-lactamases, sometimes accompanied by a decrease of outer membrane permeability, and the production of low-affinity, drug resistant Penicillin Binding Proteins (PBPs). PBPs remain attractive targets for developing new antibiotic agents because they catalyse the last steps of the biosynthesis of peptidoglycan, which is unique to bacteria, and lies outside the cytoplasmic membrane. Here we summarize the “current state of the art” of non-β-lactam inhibitors of PBPs, which have being developed in an attempt to counter the emergence of β-lactam resistance. These molecules are not susceptible to hydrolysis by β-lactamases and thus present a real alternative to β-lactams. We present transition state analogs such as boronic acids, which can covalently bind to the active serine residue in the catalytic site. Molecules containing ring structures different from the β-lactam-ring like lactivicin are able to acylate the active serine residue. High throughput screening methods, in combination with virtual screening methods and structure based design, have allowed the development of new molecules. Some of these novel inhibitors are active against major pathogens, including methicillin-resistant *Staphylococcus aureus* (MRSA) and thus open avenues new for the discovery of novel antibiotics.

## Abbreviations

PBP: penicillin binding protein
ESBL: extended spectrum β-lactamase
MRSA: methicillin-resistant strains of *Staphylococcus aureus*

## 1. Introduction

**Figure 1:**
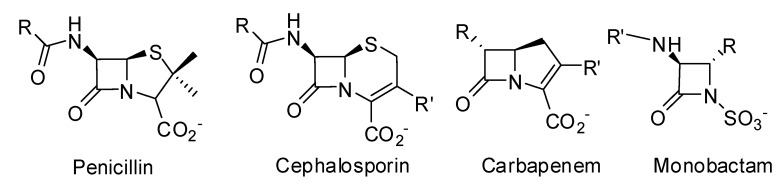
β-lactam antibiotics.

Since the discovery of penicillin by Fleming in 1928 and its clinical introduction as an antibacterial agent in the early 1950s, β-lactam antibiotics have remained the most popular drugs for treating bacterial infections. The success of penicillin led to the discovery and development of various β-lactam antibiotics: penicillins, cephalosporins, monobactams and carbapenems [1] (Figure 1), which all contain the four membered β-lactam ring. Most penicillins and cephalosporins prescribed today are chemical derivatives of the natural scaffolds produced by microorganisms [2]. Unfortunately, only 4 years after penicillin was commercialized in the early 1940s, penicillin-resistant strains of *Staphylococcus aureus* expressing and secreting a β-lactamase were isolated [3]. Some years later penicillin was found to be ineffective against a significant proportion of *S. aureus* hospital isolates [4]. The emergence of penicillin resistance led to the introduction of methicillin, a β-lactamase-insensitive semi-synthetic penicillin; but immediately after its introduction in clinical practice, methicillin-resistant strains of staphylococci (MRSA) were identified [5]. 

**Figure 2:**
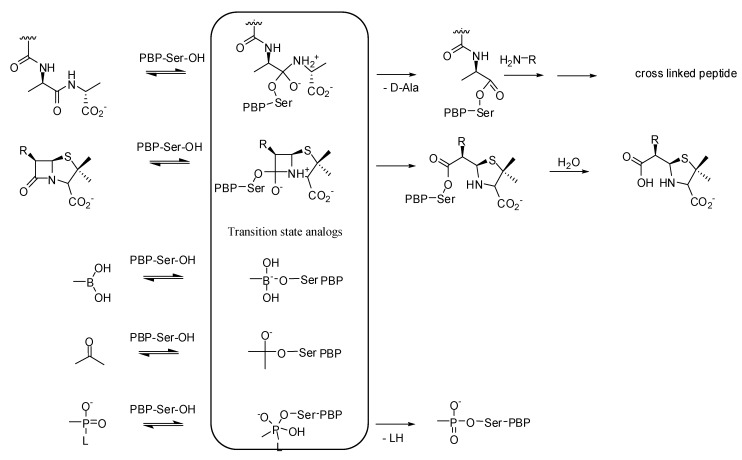
Reaction of natural substrates (peptidoglycan stem-peptides), of suicide substrates (β-lactams) and transition state analogs with reactive serine residue in the active site of PBPs.

All β-lactams share the same mode of action: they inhibit the bacterial cell wall synthesis by acting as suicide substrates of the transpeptidase domain of Penicillin Binding Proteins (PBPs). They form a stable covalent adduct with the active site serine residue of PBPs (Figure 2). The PBPs are traditionally partitioned into high molecular weight PBPs (HMW-PBPs), which are further divided in two classes, A and B, and low-molecular weight PBPs (LMW-PBPs), which are also divided in four subclasses based on their tertiary structures. HMW-PBPs are essential to cell survival and are the actual target of β-lactams. Class A PBPs catalyze the formation of the glycan chains (trans-glycosylation) and both class A and class B PBPs catalyze the cross-linking of peptidoglycan stem-peptides (transpeptidation) on the external side of the cytoplasmic membrane. Peptidoglycan is specific to bacteria and drugs which inhibit its biosynthesis have low toxicity to humans. LMW-PBPs are dispensable in laboratory conditions and thus represent minor targets of β-lactam antibiotics. 

Various mechanisms have been developed by bacteria to resist β-lactam antibiotics [6]:

- The production of β-lactamases, which catalyze the hydrolysis of the β-lactam cycle, is the most important mechanism of resistance in Gram-negative bacteria. Transfer of plasmid encoded β-lactamases rapidly disseminates resistance over a broad range of bacteria [7].
- The production of low-affinity PBPs which catalyze the transpeptidation reaction even in the presence of high concentrations of β-lactam antibiotics is an important mechanism of resistance in some Gram-positive bacteria [e.g., methicillin resistant *S. aureus* (MRSA)]. Mutation of residues surrounding the active sites of these PBPs enhances the resistance of these microorganisms to β-lactam antibiotics. Mutation of residues lowering the affinity of PBPs to β-lactams is also frequently observed in non β-lactamase producing Gram-negative bacteria and in some Gram-positive bacteria like *Streptococcus pneumoniae.* The mechanism generally affects the class-B PBPs involved in cell division (homologous to *Escherichia coli* PBP3), which is one of the main targets of β-lactams in these organisms. For example mutations are encountered in *S. pneumoniae* PBP2x [8], *Neisseria gonorrhoeae* PBP2 [9] and *Haemophilus influenzae* PBP3 [10]. Furthermore, horizontal gene transfer allows dissemination of resistance. For example, in Streptococci, resistance is disseminated via natural transformation [11], and resistance in MRSA probably originates from transduction of the *mecA* gene, coding for a methicillin-resistant PBP2a protein, into the chromosome of *S. aureus* [7,12].
- A decrease of the production of outer membrane proteins (OMPs), which allow the transfer of β-lactams through the outer membrane, lowers the effective concentration of antibiotics in the periplasm and increases MIC-values. Resistant phenotypes are observed if this mechanism is combined with another resistance mechanism such as the expression of a β-lactamase [13,14].
- In Gram-negative bacteria efflux pumps, which can export β-lactams outside the cells through the outer membrane, can also decrease the effective concentration of drugs in the periplasm [14].

Multiple strategies have been developed to fight β-lactam resistance. The search for new antibiotics and β-lactamase inhibitors has prevailed from the beginning but after sixty years of legitimate clinical utilization of antibiotics some bacterial strains have become progressively insensitive to almost all clinically useful β-lactams [15,16]. This trend has been strongly increased by misuse and overuse, including utilization as growth promoters in farm animals [17]. During the last two decades, the rapid development of resistance has discouraged pharmaceutical companies from maintaining research programs in this area, and the antibiotic discovery pipelines of most of the major companies are now nearly empty [18,19]. It is now obvious that antibiotics should be used cautiously and should be limited in the environment and food chains. These recommendations may help limiting the dissemination of resistance that is closely linked to the magnitude of the selective pressure [20]. 

**Figure 3:**
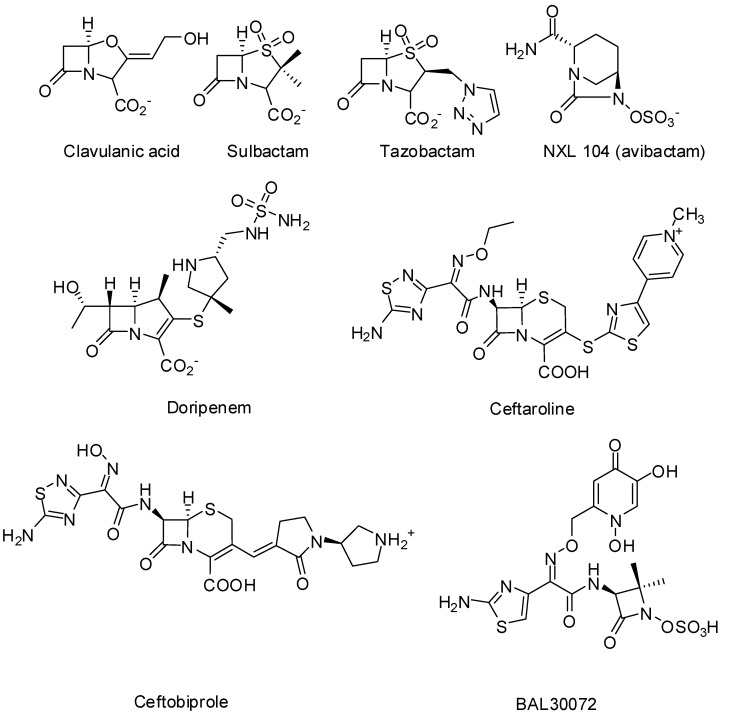
β-lactamase inhibitors and examples of the new generation of β-lactam antibiotics.

Since 1970, β-lactamase inhibitors [clavulanic acid in 1970, sulbactam in 1978 and tazobactam in 1980, (Figure 3)] have been introduced in clinical medicine [6]. They all have a four membered β-lactam ring and are inactivators or “suicide inhibitors” of class A β-lactamases. In combination with β-lactam-antibiotics (amoxicillin/clavulanate: Augmentin^TM^, ampicillin/sulbactam: Unasyn^TM^, piperacillin/tazobactam: Zosyn^TM^), they significantly lowered the MICs of the latter against various bacteria [6]. Yet, several years after the introduction of these combination drugs in clinical practice, resistance was observed, resulting from the production of inhibitor resistant β-lactamases or enzyme hyper production [6]. During the last 40 years numerous β-lactamase inhibitors, β-lactams and non-β-lactams, have been developed [6,21,22]. NXL104 (avibactam) (Figure 3) is a non-β-lactam that inhibits serine β-lactamases. In combination with extended-spectrum cephalosporins and aztreonam it is potent against Gram-negative infections (including *Klebsiella*) [23,24,25]. NXL104 has been the first β-lactamase inhibitor to be studied in clinical trials since the introduction of tazobactam [19]. 

Some new β-lactam antibiotics (carbapenems, monobactams, and cephalosporins) have been developed during the last decade or are currently in clinical trials [19,26,27]. Doripenem (Figure 3) has received FDA approval in 2007 for complicated urinary tract infections and intra-abdominal infections [19]. Doripenem is stable to hydrolysis by many β-lactamases but is hydrolyzed by class B metallo-β-lactamases and serine carbapenemases [28]. Some new carbapenems (ME1036, biapenem, panipenem, razupenem, tebipenem and tomopenem) are now in development. BAL30072 (Figure 3) is a new β-lactamase stable monobactam active against resistant β-lactamases (ESBL and metallo-β-lactamases) producing Gram-negative pathogens. Ceftobiprole (Figure 3) is a novel cephalosporin with activities against a wide range of Gram-negative (including *Pseudomonas aeruginosa*) and Gram-positive pathogens (including MRSA and penicillin-resistant *S. pneumoniae*) [29]. It is stable against some β-lactamases (non-ESBL class A) but is hydrolyzed by ESBLs and carbapenemases. Ceftobiprole is presently approved in several countries but approval in the United States was denied.

Ceftaroline (Figure 3) is a second cephalosporin approved in the USA and in the European Union for the treatment of acute bacterial skin and skin structure infections and community-acquired bacterial pneumonia [26]. It is active against the MRSA and multi-drug resistant *S. pneumoniae* and common Gram-negative pathogens [30]. In synergy with tazobactam it is active against some multi-drug resistant Gram-negative pathogens such as ESBL producing *E. coli* and *Klebsiella pneumoniae* [31]. 

All these examples show that by modifying the parent structure of traditional β-lactams the discovery of molecules active against resistant pathogens is possible. Recently, some non-traditional β-lactams (large ring 1,3-bridged 2-azetidiones) have been synthesized and some of these molecules exhibit promising activities against PBP2a of a methicillin-resistant *S*. *aureus* [32,33,34,35].

Unfortunately, some of these molecules are susceptible to hydrolysis by β-lactamases and are thus only efficient in combination with a β-lactamase inhibitor. An alternative to these molecules are non-β-lactam inhibitors of PBPs, which would not be substrates of β-lactamases. During the last three decades, efforts have been made to find non-β-lactam inhibitors, which can replace β-lactams in clinical practice. To date, only the non-β-lactam β-lactamase inhibitor NXL104 has been studied in clinical trials.

## 2. Milestones on the Way to Discover Non-β-lactam Inhibitors

Milestones on the way to discover non-β-lactam inhibitors are: (1) the availability of the target; (2) the development of an assay; (3) the choice of a strategy to find hits—new lead compounds; (4) biochemical and crystallographic studies to design new structure-based compounds; (5) *in vitro* antibacterial activities; (6) *in vivo* experiments. The milestones (3–6) will be discussed for the different non-β-lactams in the next section. 

### 2.1. Availability of the Target

Numerous PBPs from all classes including PBPs of resistant strains like PBP2a of the methicillin resistant *S. aureus* (MRSA) [36], PBP2x of penicillin resistant *S. pneumoniae* [37] and PBP5fm of drug resistant *Enterococcus faecium* [38] were cloned, overexpressed, purified, well characterized and often crystallized. 

### 2.2. Assay Development

In the past, assays have been developed to study the reaction of PBPs with β-lactams, which obeys a 3-step model:

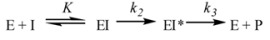
(1)
where E is the enzyme, I the β-lactam inactivator, EI the noncovalent complex and EI* the inactivated adduct or the acyl-enzyme. The acyl-enzyme is not completely stable and undergoes spontaneous hydrolysis that regenerates the active enzyme and the hydrolyzed product P. Deacylation is generally too slow to significantly influence the physiologically relevant sensitivity of a PBP to the antibiotic or the efficiency of the inactivation reaction. Since the value of *K* is rather high this sensitivity is determined by the values of the second-order rate constant *k_2_/K* [39,40].

**Figure 4:**
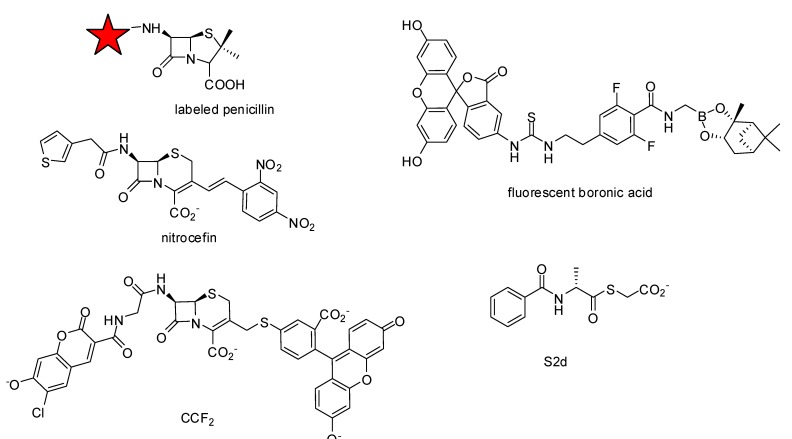
Molecules used for assay design.

Various methods have been proposed to determine *k_2_/K* and IC_50_ values. Note that IC_50_ values vary with the time of contact and the presence or absence of a pre-incubation step. Thus, they can only be compared if identical conditions are used. For very sensitive PBPs (high *k_2_/K* values) the IC_50_ can even be equal to 50% of the PBP concentration in the test sample. In some cases the formation of the acyl-enzyme can be directly monitored because the absorption [41] or the fluorescence [42,43] are modified upon β-lactam ring opening. The formation of the acylenzyme can be directly monitored with a radioactive [44], fluorescent [45,46] or biotinylated β-lactam [47] (Figure 4). For unlabeled compounds, a counter-labeling of the free enzyme can be used. Unfortunately, all these techniques require a separation of protein from the excess of reagent by gel electrophoresis, which make these methods time-consuming. Furthermore, these point by-point methods are only useful for kinetic studies if the reaction is not too fast [39]. Efforts have been done to improve these methods. Gel electrophoresis has been replaced by filtration using 96-well filter plates in assays developed for PBP2a [48]. Another approach was the immobilization of PBPs on microtiter plates by using an ELISA-like protocol [49] or a GST-PBP2a fusion protein [50]. 

A chromogenic cephalosporin, nitrocefin (Figure 4), can be used as a counter-labeling compound because its absorbance at 480–500 nm is strongly increased upon opening of the β-lactam ring [39]. The utilization of nitrocefin can directly be monitored and stopped-flow techniques can be used.

In 2012, a competition binding assay, a boronic-acid-based fluorescence polarization assay, was described that uses the reversible binding of a boronic acid “tracer” (Figure 4) and is amenable to high-throughput applications [51]. 

A thioesterase activity has been described for various PBPs of penicillin sensitive strains (PBP1b [52,53] and PBP2x both of *S. pneumoniae* [42,53], PBP3 and PBP5 both of *E. coli* [54], *Actinomadura* R39 [53] and *Streptomyces* R61 [55,56]). Assays for inhibition studies of PBP2x of a penicillin-resistant *S. pneumoniae* strains have been developed [43,53]. Thioester assays allowed a rapid screening of active compounds using 96-well microtiter plate assays [53]. Detailed kinetic studies using thioesters as reporter substrates are also possible [54,57,58]. Thioesters like S2d (Figure 4) have been used successfully for inhibition studies with hundreds of compounds in our laboratory and have shown their potential for high-throughput screening experiments.

Future assay development could use the cleavage of the β-lactam ring of cephalosporins that creates a free amino group, which triggers spontaneous elimination of any leaving group attached to the 3' position. This observation was used to detect β-lactamase activity in cells using cephalosporins with a fluorescence resonance energy transfer (FRET) pair in the R-7 and R-3 positions. Hydrolysis was followed directly by fluorescence [59,60]. Efforts have been done to use one of these molecules, the commercially available CCF2 [61] (Figure 4) in microtiter assays with various PBPs, but no spontaneous elimination of the leaving group after the formation of the acyl-enzyme was observed (personal observation).

As shown by the following models, PBP inhibitors can be classified as: (i) irreversible, covalent binding inhibitors; (ii) covalent binding inhibitors with turn-over *k_3_* (β-lactams); (iii) reversible, covalent binding inhibitors (boronic acids); (iv) non-covalent binding inhibitors (slow or rapid binding). With β-lactams the value of *k_3_* is usually low (10^−4^ M^−1^ s^−1^ or less) and this model gives results very similar to those of model (i). Moreover for many compounds (γ-lactams like lactivicin) a possible *k_3_* step has never been investigated but is also expected to be very slow:


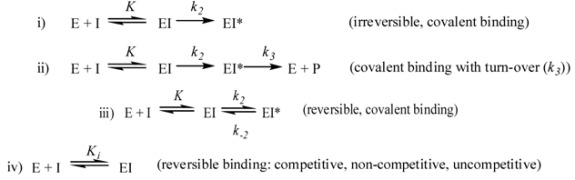


Thioester assays developed primarily for inactivators described by (ii) can be used for inhibition studies of inhibitors described by models (iii) [58] and (iv) [54]. Note that thioesters obey model (ii) but with relatively high *k_3_*-values. Competition assays using labeled β-lactams can also be used if the formation of the acyl-enzyme is not too fast and it is possible to achieve linear PBP labeling over time as described by Toney [48]. 

Now assays are available to use high-throughput screening in non-β-lactam drug discovery. A lot of time and money can be saved if assay design can be optimized to avoid the detection of new compounds which will later be identified as non-specific inhibitors. These promiscuous inhibitors plague screening libraries and hit lists. At micromolar concentrations they can form aggregates that non-specifically inhibit enzymes. These aggregates are detergent-sensitive and the addition of 0.01% Triton-X-100 in the assays can help to minimize the detection of these false positives [62,63].

## 3. Non-beta-lactams

PBPs and serine β-lactamases may be considered as serine hydrolases since they are characterized by a reactive serine side chain which makes a covalent ester bond to the carbonyl carbon atom of an amide bond (starting with either a peptide bond or a β-lactam ring) to form an acyl-enzyme. PBPs (and serine β-lactamases) and the classical trypsin and subtilisin families of serine proteases share neither sequence nor structural similarities except the sole critical involvement of a serine residue in their catalytic mechanisms. The trypsin and subtilisin families have a same catalytic triad in common (serine, histidine and aspartic acid) while PBPs have been characterized by three conserved, although not exclusively, sequence motifs in their active site (SxxK, S/YxN and K/HxG). Anyway, their basic catalytic features may be considered as common and involve a nucleophile (the serine residue), an electrophile (the oxyanion binding site) and a proton abstractor-donor (the general base).

### 3.1. Transition State Analogs

Transition state analog inhibitors (Figure 2) have been found to be efficient inhibitors of serine β-lactamases [6,21,22] as previously observed for serine proteases [64]. The overall fold of the transpeptidase domains of PBPs is similar to that of serine β-lactamases [65,66]. Screening of non-β-lactam inhibitor libraries of β-lactamases was successful and some PBP inhibitors (boronic acids and phosphonates) were identified. 

#### 3.1.1. Boronic Acids

**Figure 5:**
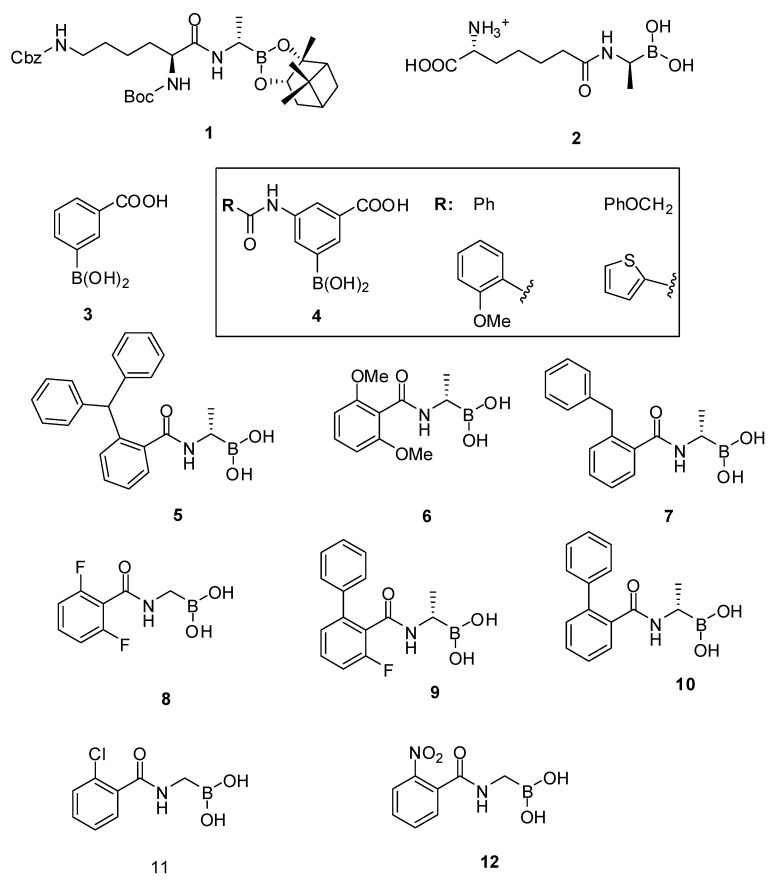
Boronic acids.

It has been known for more than three decades that boronic acids are good inhibitors of serine proteases and the considerable efforts to find inhibitors of β-lactamases have now been extended in direction of PBPs by several groups. In 2003, Pechenov *et al.* [67] developed a series of transition state analogs, amongst which they identified the peptide boronic acid Boc-L-Lys(Cbz)-*D*-boroAla **1** (Figure 5) as an excellent inhibitor of three LMW-PBPs (*N. gonorrhoeae* PBP 3: *K_i_* = 0.37 µM). 

The crystal structure of compound **1** in complex with *E. coli* PBP5 revealed, as expected, the boron covalently attached to the active serine. The complex mimics the transition-state intermediate during the deacylation step of the enzyme-catalyzed reaction [68]. More recently, the peptidyl boronic acid **2** with a diaminopimelic acid like side chain was developed to target the R39 active site. A *K_i_* value of 32 nM was obtained, which correlated well with the tight fit of the diaminopimelic side chain into the enzyme active site and the strong interactions made by this side chain ammonium and carboxylate groups with residues bordering the active site cleft, as revealed by the X-ray structure [69].

These peptidyl boronic acids certainly helped in the understanding of the underlying mechanisms of the enzyme DD-carboxypeptidase/DD-endopeptidase activity. But as they proved to be excellent inhibitors of their target PBP, further efforts were made to synthesize non-peptidyl boronic acids and assays were extended to target more medically relevant PBPs (*S. pneumoniae* PBP2x, *S. aureus* PBP2a, and *E. faecium* PBP5 [53,70,71]. 

A set of 21 commercially available boronic acids, including phenylboronic acids, thiophenyl boronic acids, an alkylboronic acid, a bicyclic benzoxaborole and a boronic acid pinacolester were tested for inhibition of R39 [70]. The most potent inhibitor was **3**, with a residual activity of 20% at 1 mM after a pre-incubation of 60 min. A library of boronic acids analogs was synthesized. Two ortho-substituted derivatives were poor inhibitors of R39 but nearly all of the meta-substituted aryl boronic acids displayed improved inhibition against R39. The most active compounds **4** have IC_50_ values in the 20–30 µM range. Some of these compounds displayed also activities against R6 PBP2x, 5204 PBP2x (penicillin resistant) and PBP1b all of *S. pneumoniae*.

**Figure 6:**
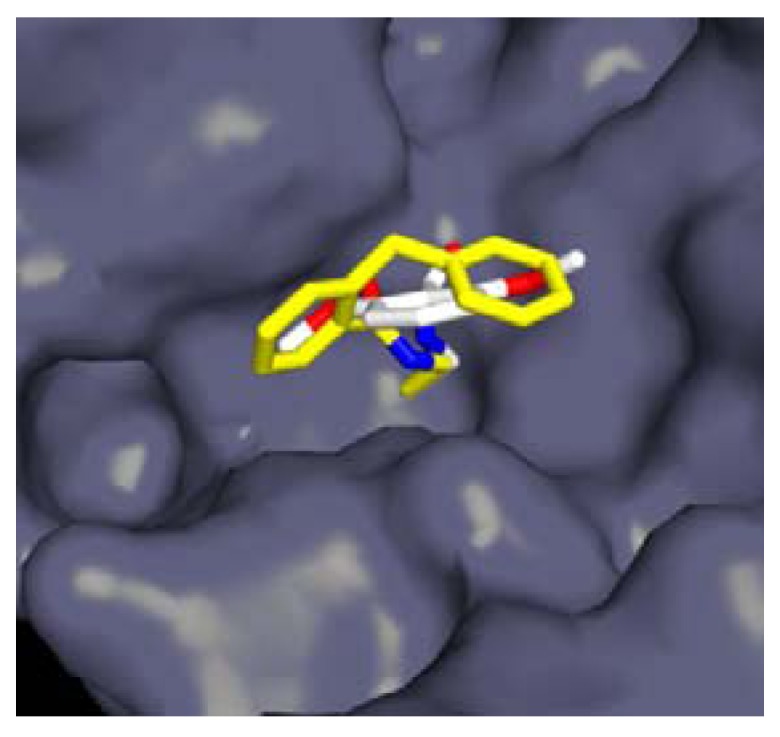
Views from a crystal structure of R39 in complex with **6** (white) and **7** (yellow). The benzyl group of **7** occupies a region predicted by computational analysis.

Amidoethylboronic acids were in general better inhibitors of R39 and structure guided development of these compounds led to inhibitors with IC_50_ values around 1 µM, the most powerful inhibitor being **5** (*K_i_* = 63 nM, IC_50_ < 0.08 µM, pre-incubation 60 min) [71]. The computer aided approach for elaborating those inhibitors from a structure of R39 in complex with **6** (IC_50_ = 33 µM, pre-incubation 60 min) was validated by the crystal structure of R39 with compound **7** (IC_50_ = 1.8 µM, pre-incubation 60 min). The structure shows both **7** aromatic rings occupying different active site pockets, which had been identified computationally and used for structural modifications of **6** (Figure 6). 

Some amidomethyl- and amidoethylboronic acids were inhibitors of *S. pneumonia*e PBP1b with IC_50_ values lower than 20 µM, the most powerful inhibitors being **5** (IC_50_ = 4.9 µM, pre-incubation 60 min) and **8** **(**IC_50_ = 6.9 µM, pre-incubation 60 min). A series of crystal structures of amidoethyl- and amidomethylboronic acids could also be obtained with *S. pneumoniae* PBP1b, some of these inhibitors showing antibacterial activity against methicillin resistant *S. aureus* (Table 1) [52]. 

**Table 1:** Minimal inhibitory concentrations MIC (µg/mL) of amidoethylboronic acids.

Organism	Compound	
	9	10
*Bacillus subtilis* ATCC 6633	16	32
*Listeria monocytogenes* ATCC 14780	32	64
*Enterococcus hirae* ATCC 8790	32	32
*Staphylococcus aureus* ATCC25923	32	64
*Staphylococcus aureus* ATCC 43300 (MRSA)	32	128

**Figure 7:**
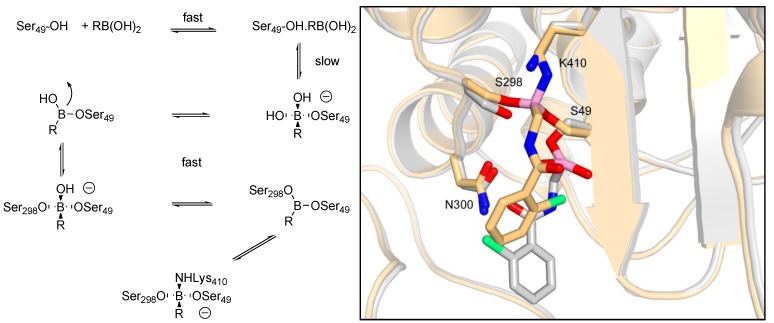
Reaction scheme and overlap of monocovalent adduct (white) and tricovalent adduct (gold) of **11** with R39. Oxygen atoms are coloured red, nitrogen blue, boron pink and chlorine green [58].

The structures revealed two distinct side chain binding modes: the methyl group of the *S*-amidoethyllboronic acids (**5**–**7**, **9** and **10**) bound in the active site region similarly to the amide side chain of penicillins and cephalosporins, and thus should be more representative of substrate analogs than the amidomethylboronic acids or the *R*-amidoethylboronic acids, the side chain of which could bind in an alternative region of the enzyme catalytic cleft. This was even more dramatically observed in structures of R39 in complex with amidomethylboronic acids [58]. The boron atom covalently binds to the active serine Ser49 forming a monocovalent adduct but the ligand further inserts into the active site so that the boron is linked to one lysine Lys410 and two serine residues Ser49 and Ser298 and eventually makes a tricovalent adduct with the enzyme (Figure 7). On the basis of the crystallographic and kinetic results, a reaction scheme for this inhibition by boronic acids was proposed (Figure 7). 

*S*-Amidoethylboronic acids display a greater analogy to the natural substrate than *R*-ethyl- or methyl-boronic acids. In R39 as well as in PBP1b, the Cα methyl group inserts into a hydrophobic pocket that plays an important role in the substrate specificity toward the D-alanine as the penultimate residue of the peptidoglycan stem pentapeptide. Thus boronic acids with larger substituent on the Cα methyl group should be very poor inhibitors of PBPs, as was shown in the case of R39 and PBP1b. In both enzymes, the binding modes that are not genuine analogs of substrate binding could represent an interesting alternative to find specific inhibitors; 2-nitrobenzamidomethylboronic acid **12** represents such an inhibitor of R39 [58].

Boronic acids are rarely used in medicinal chemistry [72]. The experimental evidence of their great PBPs inhibitory potential should encourage future developments and prospects as the fight against bacterial resistance to β-lactams continues. 

#### 3.1.2. Carbonyl Compounds

**Figure 8:**
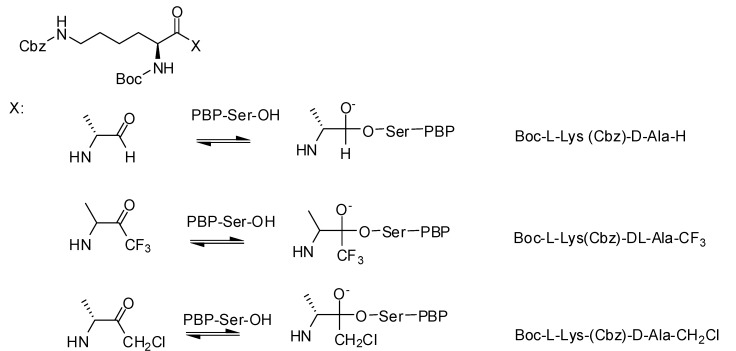
Carbonyl compounds.

Carbonyl compounds have been systematically studied by Pechenov *et al.*, who synthesized peptide chloromethyl ketones, trifluoromethyl ketones and aldehydes as potential transition state analogs. Boc-L-Lys(Cbz)-D-Ala was chosen as the parent structure, since it is a simple dipeptide mimic of the natural PBP substrate (Figure 8) [67]. 

The peptide aldehydes Boc-L-Lys(Cbz)-D-Ala-H (*Ki* = 60 µM) and Boc-L-Lys(Cbz)-L-Ala-H (*Ki* = 79 µM) were identified as inhibitors of *N. gonorrhoeae* PBP3. A *Ki*-value of 60 µM was described for the inhibition of the same PBP3 by the diastomeric mixture of Boc-L-Lys(Cbz)-D,L-Ala-CF_3_ while the chloromethyl ketone showed no inhibitory activity on PBPs. Trifluoromethylketone analogs of good boronic acid inhibitors of *Actinomadura* R39 were studied but showed no inhibitory activity [71].

#### 3.1.3. Phosph(on)ates

**Figure 9:**
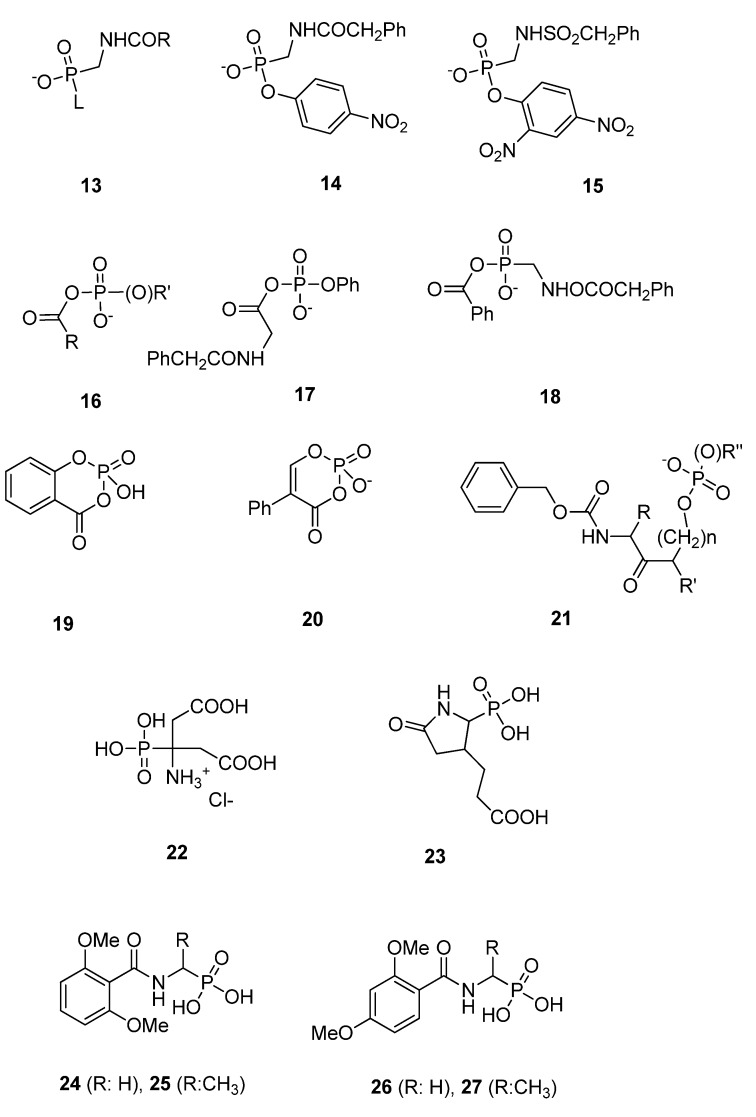
Phosph(on)ates.

Neutral phosphyl reagents are strong inhibitors of serine proteases [64]. Since the 1980s various phosph(on)ates were synthesized as inhibitors of β-lactamases. Often the DD-peptidase of *Streptomyces* R61 was used as a model enzyme to explore the inhibitory potential of phosph(on)ates on PBPs. Phosphonate monoesters **13** (Figure 9) were found to be the first β-lactamase inhibitors [73,74,75]. After formation of a trigonal bipyramidal transition state and departure of the leaving group L, β-lactamases form stable tetrahedral phosphonyl-enzyme adducts with phosphonate monoesters (Figure 2) [76,77,78]. The inactivation can be described by the inhibition model (ii) shown above. The formation of the covalent adduct, the acyl phosphonate, is characterized by a second-order rate constants *k_2_/K*. The stability of the E-I complex is characterized by the rate constant *k_3_*. Inhibitory power of phosphonate monoesters can be improved by selection of a good leaving group L and by modification of the amido side chain R. The phosphonate monoesters **14** and **15** (Figure 9) were poor inhibitors of R61 with *k_2_/K-*values of 0.07 M^−1^s^−1^ [75] and 0.06 M^−1^s^−1^ [73], respectively*.* The observation that leaving group lability is an important element led to the development of acyl phosph(on)ates **16** (Figure 9). These molecules can form both acyl- and phosphor(on)yl-enzyme species. The acyl phosphate **17** was found to be a substrate of typical class A and class C β-lactamases and a good substrate of R61 (*K_m_* = 0.2 mM, *k_ca_*_t_ = 4.1 s^−1^). The acyl phosphonate **18** was an irreversible inhibitor of β-lactamases, probably by phosphonylation of the serine (Figure 9), but was found to be a poor inhibitor of R61 with *k_2_/K-*value of 5 × 10^−3^ M^−1^s^−1^. A very slow turnover of this molecule by R61 was observed (*K_m_* = 0.21 mM, *k_ca_*_t_ = 3.5 × 10^−4^ s^−1^) meaning that an acyl-enzyme is formed or that the phosphoronyl enzyme is unstable [79]. 

Cyclic variants of phosph(on)ates like **19** were proposed. In this case the leaving group actually does not leave and avoids hydrolysis and rapid regeneration of the free enzyme [80]. The crystal structure of R61 with compounds **19** and **20** revealed that the R61 enzyme is preferentially phosphorylated rather than acylated. Both molecules inactivated R61 rather slowly (*k_2_/K-*values of **19** and **20**: 0.46 M^−1^s^−1^ and 24 M^−1^s^−1^, respectively) and form long-lived intermediates (*k_3_*-values of **19** and **20**: 2.7 × 10^−3^ s^−1^ and 8.9 × 10^−3^ s^−1^, respectively) [81]. Amidoketophosph(on)ates **21** inhibit typical class C and class D β-lactamases but not R61 [82].

Diisopropyl fluorophosphate (DIFP), which is efficient against serine proteases, was a poor inhibitor of *E. coli* PBP 5 (residual activity of 72% at a concentration of 1 mM after a pre-incubation of 1 h) [83]. A rather poor inhibition of *Actinomadura* R39 by phosphonic bioisoster of aminocitrate (**22**, residual activity of 47% at a concentration of 500 µM) and pyrrolidinone (**23**, residual activity of 67% at a concentration of 500 µM) (Figure 9) was described [84]. The enzymatic assay with R39 was done using a thioester assay [53] and a pre- incubation time of one hour (it should be noted that the experimental details described in the original publication—fluorescent ampicillin and a 16 h pre-incubation—are not correct). The phosphonates **24**–**27** show a modest inhibition of R39 (residual activity of 86%, **24** and **25**, residual activity of 65%, **26** and **27** residual activity of 76%, all at a concentration of 1 mM and a pre-incubation of 1 h, respectively), [71] whereas the corresponding boronic acid of **24** [58] and **25** are good inhibitors of R39. The boronic acid analogue of **26** was a poor inhibitor (residual activity of 69% at a concentration of 1 mM). [53] These results probably reflect the structural differences between the covalent complexes formed between the enzyme and the boronic acids and the phosphonates, respectively. In phosphonate adducts the 2,4 dimethoxybenzoylamino side chain fits probably better than the 2,6 dimethoxybenzoylamino chain in the active site. Compounds **22**–**27** contain two hydroxyl groups which are poor leaving groups. The inhibitory power of the published compounds could probably be optimized by the introduction of a good leaving group and by the modification of the amido side chain. A crystal structure of a PBP-phosphonate complex is awaited to develop a structure based design of potent phosphonate inhibitors.

### 3.2. Substrate Analogs

**Figure 10:**
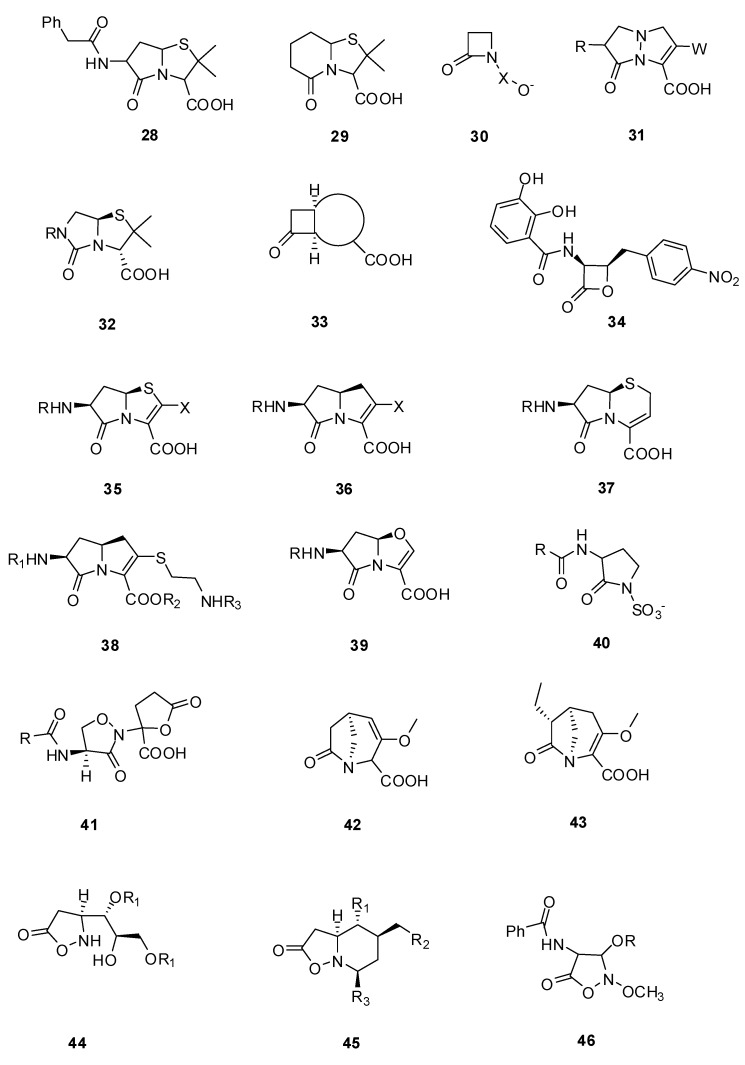
Substrate analogs.

This paragraph describes molecules that can react as suicide substrates by acylation of the PBP active serine, similarly to acylation by β-lactams. In the 1940s, the first γ-lactam **28** and δ-lactam **29** (Figure 10) mimics of penicillin have been prepared but these compounds were biologically inactive. From the 1970s efforts were done by different groups and companies to replace the β-lactam ring. By the early 1980s the intensive research on β-lactams allowed the simplification of the complex structures to a sufficiently reactive pharmacophore, the azetidinone **30** (Figure 10) [85] which guided further drug development. This molecule possesses the correct molecular shape for binding to the target PBPs. The presence of an acidic group with a distance of about 3.0–3.6 Å between the lactam carbonyl carbon and the acidic group center is essential. The “correct molecular shape” is not sufficient; a suitable reactivity of the lactam moiety is also required. The reaction with hydroxide was used as a model to study the chemical reactivity of lactams showing that some biological inactive compounds like **28** were not hydrolyzed by hydroxide at room temperature [86]. Details on the synthesis of non-β-lactam mimics are summarized in two reviews [87,88].

In 1992, the initial examples of biologically active compounds were summarized by Jungheim and Ternansky [85]. Until the early 1990s various non-β-lactam structures have been studied: pyrazolidinones **31**, imidazolidinones **32**, cyclobutanones **33**, β-lactones like obafluorin **34**, isolated from bacterial cultures and γ-lactams (analogs of penems **35**, of carbapenems **36**, of 3-unsubstituted cephems **37**, of carbapenems **38**, of oxapenems **39** and of monobactams **40**) and isoxazolidinones like lactivicins **41**. From these compounds, only two classes clearly inhibited PBPs and exhibited clinically relevant levels of antibacterial activities: the bicyclic pyrazolidinones **31** and the lactivicins **41**, which will be discussed in detail. All other compounds were inactive or exhibited poor biological activities [85,87,88]. 

In 2004, the bridged γ-lactams **42** and **43** (Figure 10) were studied [89]. These molecules were chemically unstable with half-lives of 14 and 25 min respectively and showed weak antibacterial activities against Gram-positive and Gram-negative bacteria. In 1998 isoxazolidin-5-one analogs of β-lactam antibiotics **44** and **45** (Figure 10) were synthesized [90]. Some of these molecules have low *in vitro* antimicrobial activity against *E. coli* and *S. aureus.* Isoxazolidin-5-one analogs **46** (Figure 10) had good biological activities against *Bacillus subtilis* (MIC 0.2 to 10 µg/mL depending of residue R) [91]. These molecules bound irreversibly to PBPs and were inhibitors of class A, B and D β-lactamases. 

In 2000 Imming *et al.* studied the hydrolytic stability *versus* ring size in lactams. They proposed the use of δ-valerolactam as a promising starting point for the development of a new class of lactam antibiotics because of its high reactivity similar to that of β-propiolactam [86]. This area remains to be explored. The most exciting molecule is actually NXL104 (Figure 3), a bridged bicyclo[3.2.1]-diazabicyclooctanone, which inactivates class A and class C β-lactamases at nanomolar concentrations but has no activities on PBPs. This molecule is presently in clinical trial [92]. Some related compounds like NXL105 are described which are not only β-lactamase inhibitors but have also some antibacterial activities mostly against *P*. *aeruginosa* [23]. 

#### 3.2.1. Pyrazolidinones

Biological active prototypes of bicyclic pyrazolidinones **47** (Table 2) were designed at the Lilly Research laboratories [93]. Modification of the substituents at C-3 (W) and C-7 (R) of **31** (Figure 10) allowed the variation of biological activities. Side chains from active β-lactam compounds like the ATMO—aminothiazoylmethoxime-side chain were introduced in C-7 position in **48** and **49** (Table 2). Compounds like **48** and **49** with strong electron-withdrawing groups in C-3 position were shown to be more sensitive to hydroxide ions and had better *in vitro* activities than the original prototype **31** [85]. 

**Table 2:** Minimal inhibitory concentrations MIC (µg/mL) of pyrazolidinone analogs [85]. 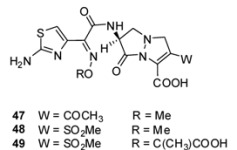

Organism	Compound		
	47	48	49
*Staphylococcus aureus (X1.1)*	32	32	>128
*Streptococcus pyogenes (C203)*	0.5	0.13	1
*Haemophilis influenzae (76)*	8	0.5	0.03
*Escherichia coli (EC14)*	2	0.06	0.03
*Klebsiella pneumoniae (X26)*	2	0.13	0.13
*Enterobacter cloacae (EB5)*	16	0.25	0.13

#### 3.2.2. Lactivicin Analogs

In 1986, lactivicin (LTV) **50** (Table 3) was isolated from bacterial strains (*Empedobacter lactamgenus* and *Lysobacter albus*) by the Takeda Research group [94,95,96]. LTV, which has a unique ring structure comprising a functionalized L-cycloserinyl ring linked to a γ-lactone ring, was the first natural PBP inhibitor without a β-lactam ring. LTV is active against a wide range of Gram-negative bacteria and highly active against Gram-positive bacteria. LTV derivatives, where the 4-aminolactivicinic acid LTV nucleus was acylated by various residues, were synthesized to increase its antibacterial activity against Gram-negative bacteria and to overcome its relatively strong toxicity (Table 3) [87,97,98,99]. 

**Table 3:** Minimal inhibitory concentrations MIC (µg/mL) of lactivicin analogs [87]. 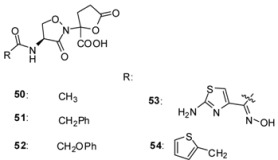

Organism	Compound				
	50	51	52	53	54
*Staphylococcus aureus* (FDA 209P)	3.1	0.2	0.4	1.6	0.4
*Escherichia coli* (O111)	100	3.1	6.3	1.6	3.1
*Klebsiella pneumoniae* (DT)	100	3.1	25	3.1	6.3

**Figure 11:**
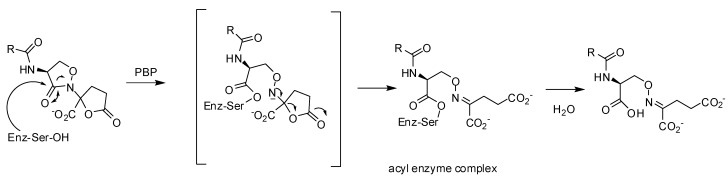
Reaction of lactivicin in the active site of *S. pneumoniae* PBP1b [100].

Crystallographic analyses of *S. pneumoniae* PBP1b with LTV and a more potent analog, phenoxyacetyl-lactivicin (PLTV, **52**) reveal that inhibition of PBPs involves the opening of both monocyclic cycloserine and γ-lactone rings and the formation of a stable covalent adduct with the active site serine (Figure 11). PLTV **52** was a more efficient inactivator of 5204 PBP2x of penicillin resistant *S. pneumoniae* with an IC_50_-value of 7.9 µM (pre-incubation: 60 min) compared to 150 µM (pre-incubation: 120 min) for LTV **50**. PLTV showed antimicrobial activity against drug-sensitive (MIC: 2 µg/mL) and various drug-resistant *S. pneumoniae* strains (MIC: 10 or 20 µg/mL) [100]. 

### 3.3. Non-covalent Inhibitors

#### 3.3.1. Arylalkylidene Rhodanines and Arylalkylidene Iminotriazolidenes

**Figure 12:**
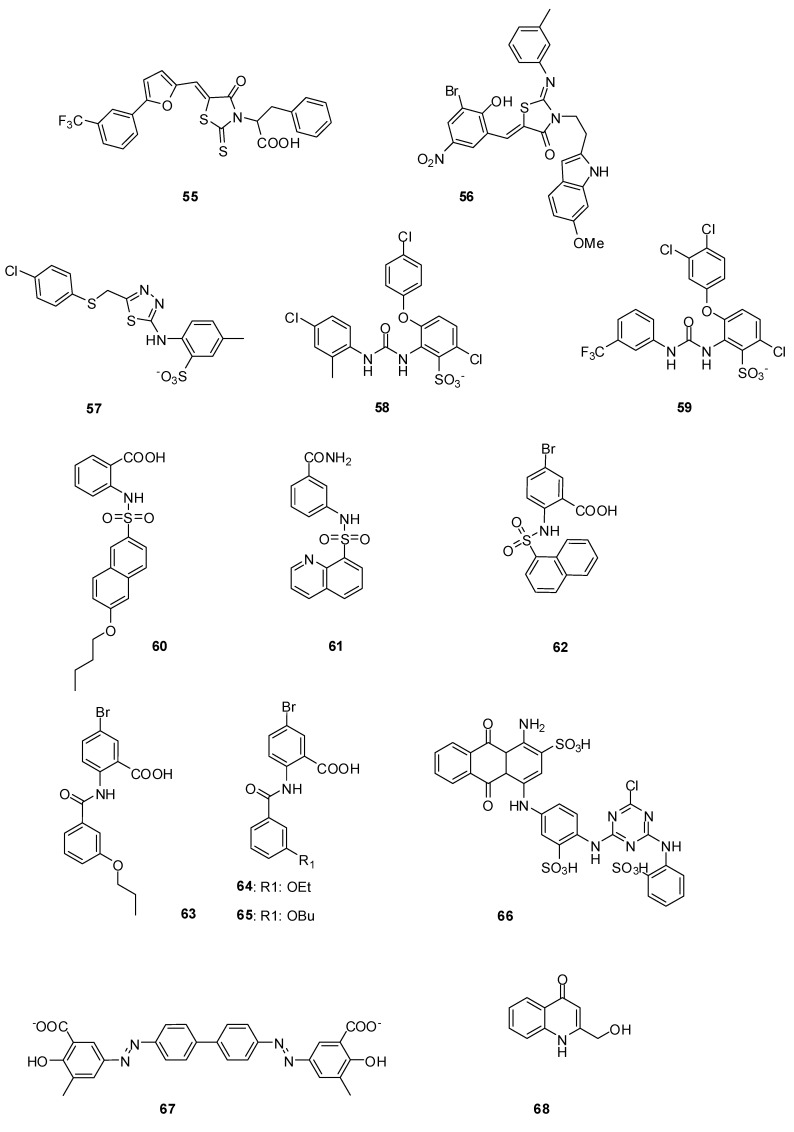
Non-covalent inhibitors.

A screening of chemical libraries led to a series of rhodanines, which were found to be inhibitors of class C β-lactamases in the micromolar range [101]. Similar compounds were found to be inhibitors of various PBPs including PBP2x of a penicillin resistant strain of *S. pneumoniae* and *S. aureus* PBP2a*.* Furthermore *in vitro* activities against various bacterial strains including *S. aureus* (MRSA), *S. pneumoniae* (PRSP), and *E. faecium* (VRE) strains were described. A detailed kinetic study with the most active compounds **55** and **56** (Figure 12) shows that the inhibitor activity of these slow-binding inhibitors is non-competitive, which means that they probably do not bind to the active site of β-lactam binding enzymes [54]. The inhibition by compound **55** is detergent-sensitive and inhibition activity cannot be detected in presence of 0.01% Triton-X-100 (personal communication) underlining that the detection of non-competitive inhibitors -promiscuous inhibitors- can be avoided by addition of detergents in the enzymatic assay as previously mentioned.

#### 3.3.2. Aminothiadiazole and *Ortho*-phenoxyldiphenylurea Derivatives

Aminothiadiazole derivate **57** and two ortho-phenoxyl diphenylurea compounds **58** and **59** are inhibitors of R6-PBP2x and 5204-PBP2x both of *S. pneumoniae* strains [R6 (penicillin sensitive) and 5204 (penicillin resistant)], Table 4, Figure 12 [102]. 

**Table 4:** Inhibi*t*ion of PBPs of resistant strains: PBP2a of methicillin resistant *S. aureus*, PBP5fm of drug resistant *E. faecium* D63r and 5204 PBP2x of penicillin resistant *S. pneumoniae*.

Molecule	PBP2aIC_50_ [µM]	PBP5fmIC_50_ [µM]	5204 PBP2xIC_50_ [µM]
57 ^1^	nd	nd	219
58 ^1^	nd	nd	71
59 ^1^	nd	nd	72
60 ^1^	97	no inhibition at 1 mM	391
61 ^1^	residual activity *: 58%	930	no inhibition at 1 mM
62 ^1^	80	no inhibition at 1 mM	nd
63 ^1^	230	residual activity *: 72%	155
64 ^1^	490	residual activity *: 83%	nd
65 ^1^	352	residual activity *: 85%	nd
66 ^2^	24	nd	nd
67 ^2^	13	nd	nd

* at a concentration of 1 mM, ^1^ assays done in the presence of 0.01% Triton-X-100 and a pre-incubation of 4 h. ^2^ pre-incubation of 60 min.

The discovery of these compounds was the result of a structure-based virtual screening. A pharmacophore model developed from an in-house database of active compounds against PBP2x was used to screen the NCI database containing 260,071 compounds. 

#### 3.3.3. Naphthalene Sulfonamides

The development of high throughput screening assays for PBPs has allowed the screening of compound libraries. After a screening of an in-house bank of 250 compounds from different non-reactive chemical classes, **60** (Figure 12) was identified as an initial hit, which inhibited PBP2a with a -positive strains including *E. faecium* ATCC 19434 (MIC: 64 µg/mL), various *S. pneumoniae* promising IC_50_ of 97 µM, and 5204 PBP2x with a not less interesting IC_50_ of 391 µM (Table 4). It also showed *in vitro* antibacterial activities against some Gram strains (MIC: 1 µg/mL), as well as both penicillin sensitive and resistant *S. aureus* strains (MIC: 32 µg/mL). A small library of structurally related compounds was obtained by performing computational similarity searches based on the structure of **60** as a starting point and using the ChemBridge bank of compounds containing more than 800,000 compounds [103]. From a series of naphthalene sulfonamides (four compounds), **61** (Figure 12) was a moderate inhibitor of PBP5fm but had no significant antibacterial activity. Various naphthalene sulfonamides were synthesized by the same authors to clarify the structure-activity relationship for PBP inhibition. Some inhibitors of PBP2a were found with IC_50_-values in the micromolar range. The best inhibitor was **62** (Figure 12, Table 4). Unfortunately all naphthalene sulfonamides described in this study were only poor inhibitors of bacterial growth [104].

#### 3.3.4. Anthranilic Acids

5-Bromo-2-(3-propoxybenzamido) benzoic acid (**63**) is a promising anthranilic acid inhibitor of PBP2a and 5204 PBP2x (Figure 12, Table 4). This compound showed a good antibacterial activity against Gram-positive bacterial strains, including *E. faecium* ATCC 19434 (MIC: 16 µg/mL), various *S. pneumoniae* strains (MIC: 1 µg/mL), as well as both sensitive and resistant *S. aureus* (MIC: 32 µg/mL). **63** was detected by performing computational similarity searches based on the structure of an analogue of **60**, where the sulfonamide bond was replaced by an amide, as already described in paragraph 3.3.3 [103]. Chemical synthesis of a library of anthranilic acid analogs of **63** allowed the discovery of inhibitors of PBP2a in the micromolar range. The best of these inhibitors were **64** and **65** (Figure 12, Table 4). Both compounds showed good antibacterial activities against *Listeria innocua*, *L. monocytogenes*, *S. epidermidis* and *B. subtilis* strains, with MICs of 4 µg/mL or 8 µg/mL. Furthermore with **65**, the growth of two MRSA strains, with MICs of 4 µg/mL and 2 µg/mL, was 16- to 32-fold more efficiently prevented than that of the penicillin-sensitive *S. aureus* strain, which had a MIC of 64 µg/mL. The author suggested that compounds **64** and **65** exert their *in vitro* antibacterial activities by targeting other proteins besides their inhibition of PBPs, because the MIC values are significantly lower than the IC_50_ ones. 

#### 3.3.5. Cibacron Blue and Erie Yellow

Cibacron Blue **66** and Erie Yellow **67** (Figure 12, Table 4) were identified as inhibitors of PBP2a, with IC_50_-values of 24 µM and 13 µM, respectively [48]. The screening was done without addition of detergents like Triton-X-100 or the presence of bovine serum albumin to avoid the detection of promiscuous inhibitors. The kinetics of the inhibition mechanism were not studied and no crystallographic data are available. It is unclear whether these molecules competitively bind to the active site. 

#### 3.3.6. Cyclic Peptide

A disulfide bond containing cyclic heptapeptide (sequence NH_2_-CYHFLWGPC-COOH), first selected as a β-lactamase inhibitor, was described as an inhibitor of some PBPs [105]. Competition experiments with fluorescent ampicillin were used to measure IC_50_-values in the micromolar range. The kinetic mechanism of inhibition and the influence of detergents were not studied. In the absence of crystallographic data, it is not clear if this peptide binds competitively to the active site.

#### 3.3.7. 4-Quinolones

4-Quinolones were found to be noncovalent inhibitors of PBPs of *E. coli* and *B. subtilis*. The initial lead, an inactive 4-quinolone, was composed from fragments and docked into the active site of *E. coli* PBP5. Series of 4-quinolones were designed and synthesized. Dissociation constants (*K_i_*) with membrane-bound PBPs of *E. coli* in the micromolar range were detected and inhibiton of PBPs of *B. subtilis* was observed. The most efficient binding was observed with compound **68** (Figure 12), with *Ki* values around 30 µM for each high molecular mass PBP of *E. coli* (PBP1a/1b, PBP2 and PBP3). All active 4-quinolones had no *in vitro* antibacterial activities against *E. coli* or *B. subtilis* [106].

## 4. Conclusions

The “golden age” of antibiotics has been over for several years since usually, as new drugs are introduced, only a short time elapses before resistance emerges. In this context, identification of new innovative drugs is highly needed. Some promising non-β-lactam molecules have been discovered representing lead-structures for the development of new antibiotics. Considerable efforts were done 30 years ago to find biological active pyrazolidinones and lactivicin analogs by chemical intuition and structure-activity relationship studies. Nowadays crystallization of various PBPs is more accessible and has recently allowed the elucidation of the inhibition mechanisms of lactivicin and boronic acids. In recent studies of some of these “old” structures like lactivicin, the authors have described their activities against clinically isolated penicillin resistant *S*. *pneumoniae* strains. Starting from crystal complexes computer aided drug design is a powerful instrument to find more potent inhibitors as was recently shown for amidoethylboronic acids. The combination of high throughput screening methods in combination with virtual modeling has allowed the discovery of some new molecules, which are active against clinically important pathogens like MRSA. Now, the antibacterial drug discovery field takes advantage of the contribution of new methodological approaches and strategies like screening for synthetic inhibitors by targeted approaches including structure-based design, the search for new natural product leads from different sources and analyses of focused libraries. Yet powerful instruments like high throughput screening methods, crystallization protocols for various PBPs and virtual modeling programs are available, which could be used to optimize “old structures” or to find new lead-structures in the future. Unfortunately, most of the pharmaceutical companies have neglected to invest in antibiotic research and very few of them have spent efforts and money on the development of new classes of antibiotics. There is no time to waste and the scientific world and governments have to find ways to convince pharmaceutical companies to invest more in antibiotic research.
